# Nocturnal Hypoventilation in the Patients Submitted to Thoracic Surgery

**DOI:** 10.1155/2023/2162668

**Published:** 2023-08-09

**Authors:** Maciej Majchrzak, Cyryl Daroszewski, Piotr Błasiak, Adam Rzechonek, Paweł Piesiak, Monika Kosacka, Anna Brzecka

**Affiliations:** ^1^Department of Thoracic Surgery, Wrocław Medical University, Wrocław 53-439, Grabiszyńska 105, Poland; ^2^Department of Pulmonology and Lung Oncology, Wrocław Medical University, Wrocław 53-439, Grabiszyńska 105, Poland

## Abstract

**Introduction:**

Nocturnal hypoventilation may occur due to obesity, concomitant chronic obstructive pulmonary disease (COPD), obstructive sleep apnea, and/or the use of narcotic analgesics. The aim of the study was to evaluate the risk and severity of nocturnal hypoventilation as assessed by transcutaneous continuous capnography in the patients submitted to thoracic surgery.

**Materials and Methods:**

The material of the study consisted of 45 obese (BMI 34.8 ± 3.7 kg/m^2^) and 23 nonobese (25.5 ± 3.6 kg/m^2^) patients, who underwent thoracic surgery because of malignant (57 patients) and nonmalignant tumors. All the patients received routine analgesic treatment after surgery including intravenous morphine sulfate. Overnight transcutaneous measurements of CO_2_ partial pressure (tcpCO_2_) were performed before and after surgery in search of nocturnal hypoventilation, i.e., the periods lasting at least 10 minutes with tcpCO_2_ above 55 mmHg.

**Results:**

Nocturnal hypoventilation during the first night after thoracic surgery was detected in 10 patients (15%), all obese, three of them with COPD, four with high suspicion of moderate-to-severe OSA syndrome, and one with chronic daytime hypercapnia. In the patients with nocturnal hypoventilation, the mean tcpCO_2_ was 53.4 ± 6.1 mmHg, maximal tcpCO_2_ was 59.9 ± 8.4 mmHg, and minimal tcpCO_2_ was 46.4 ± 6.7 mmHg during the first night after surgery. In these patients, there were higher values of minimal, mean, and maximal tcpCO_2_ in the preoperative period. Nocturnal hypoventilation in the postoperative period did not influence the duration of hospitalization. Among 12 patients with primary lung cancer who died during the first two years of observation, there were 11 patients without nocturnal hypoventilation in the early postoperative period.

**Conclusion:**

Nocturnal hypoventilation may occur in the patients after thoracic surgery, especially in obese patients with bronchial obstruction, obstructive sleep apnea, or chronic daytime hypercapnia, and does not influence the duration of hospitalization.

## 1. Introduction

With increasing global epidemics of obesity [1, 2] and the progress in thoracic surgical procedures [3], the probability of a need to perform surgical treatment of lung tumors in obese patients also increases. It has been suggested that the obese patients submitted to thoracic surgery should be distinguished as a special group of patients at the increased risk of perioperative complications [4]. Obesity itself carries a risk of occurrence of abnormalities in the breathing pattern, especially during sleep [5]. Sleep hypoventilation is one of the sleep-breathing disorders occurring in some of the obese persons [6].

Sleep hypoventilation as assessed by transcutaneous continuous measurement of carbon dioxide partial pressure (tcpCO_2_) may be defined as an increase of tcpCO_2_ to a value >55 mmHg for a period of at least 10 minutes or an increase of tcpCO_2_ to a value >50 mmHg, but also exceeding by ≥10 mmHg awake values, for a period of at least 10 minutes [7].

In obese patients, sleep hypoventilation may occur even in the absence of diurnal hypercapnia [8]. It may be regarded as a sign preceding the development of chronic respiratory insufficiency [8].

Beyond obesity, the other main causes of nocturnal hypoventilation include chronic obstructive pulmonary disease (COPD), obstructive sleep apnea (OSA), neuromuscular disorders, and severe chest wall abnormalities [9, 10]. In COPD patients, hypoventilation during sleep is a common feature, first appearing during REM (rapid eye movement) sleep; then, in non-REM sleep, before daytime, chronic hypercapnia will develop [11]. OSA, although very common in the adult population, in majority of cases remains unrecognized and untreated [12]. In addition, OSA frequently co-occurs with obesity and, in minor degree, with COPD [10].

Sleep hypoventilation, causing nocturnal hypoxemia and hypercapnia, is not rarely severe [13] and may be aggravated by the suppression of central respiratory drive, such as occurring after opioid analgesics [14]. Opioids, in turn, are routinely ordered after thoracic surgical procedures [15]. On the other hand, postoperative pain has to be managed properly, and the doses of opioid use have to be sufficient in order to avoid suffering of the patient [16]. Up to 46% of patients may experience opioid-induced hypoventilation in the postoperative period [17]. This is particularly important as the patients with postoperative hypoventilation are at 2.5 times increased risk of rescue actions [17]. This might be important especially in the patients undergoing surgical procedures that diminish the area of respiratory gas exchange. Thus, the patients undergoing pulmonectomy, lobectomy, or anatomical wedge resection might be at risk of developing respiratory insufficiency during sleep after thoracic surgery due to sleep hypoventilation.

Regardless of the cause of nocturnal hypoventilation during sleep, it may be associated with an increased risk of postoperative morbidity [18]. It has been found that the patients with hypoventilation during sleep might need increased caution and care during the postoperative period [19].

The frequency of occurrence of nocturnal hypoventilation in the patients submitted to thoracic surgery has not been established yet. In addition, it is still unknown whether the nocturnal hypoventilation in the patients after thoracic surgery is associated with prolonged hospitalization or remote consequences.

The aim of the study was to evaluate the risk of the occurrence of sleep hypoventilation as assessed by whole-night capnography, in the postoperative period in the surgically treated obese and nonobese patients with malignant and nonmalignant tumors of the lung, and to assess the two-year survival in the lung cancer patients with and without sleep hypoventilation.

## 2. Materials and Methods

Material of the study encompassed 68 patients (36 women and 32 men), aged 40–83 years, with a mean age of 64.1 ± 7.7 years. The body mass index (BMI) of the patients ranged from 15.6 kg/m^2^ to 44.1 kg/m^2^, and the mean BMI was 31.6 ± 5.7 kg/m^2^. There were 45 obese (BMI ≥ 30 kg/m^2^) patients (66%), 16 overweight (BMI 25–29.9 kg/m^2^) patients (24%), 5 normal weight (BMI 20–24.9 kg/m^2^) patients (7%), and two underweight (BMI < 20 kg/m^2^) patients (3%). Spirometric studies were performed in all the patients before surgery. COPD was diagnosed when the FEV_1_ (forced expiratory volume in one second)/FVC (forced vital capacity) ratio after inhaled bronchodilator was <70%. The stages of COPD were classified as follows: stage 1, when FEV_1_ was ≥80%; stage 2, when FEV_1_ was between 50% and 79%; stage 3, when FEV_1_ was between 30% and 49%; and stage 4, when FEV_1_ was <30% of predicted [20]. There were 36 patients (55%) with COPD, including 19 patients in stage 1, 16 patients in stage 2, and one patient in stage 3, and there were no patients in stage 4. In all the patients, arterial blood gas (ABG) studies were performed before surgery and repeated a day after surgery. Chronic daytime hypoventilation (PaCO_2_ > 50 mmHg during wakefulness) was found in one patient. None of the patients used opioids in the preoperative period.

Thoracotomy was performed in 43 patients (63%), including pulmonectomy in five patients, lobectomy in 24 patients, wedge resection in 12 patients, and no lung tissue removal in two patients (hiatal hernia). Video-assisted thoracic surgery (VATS) was performed in 25 patients (37%), including pulmonectomy in 2 patients, lobectomy in 14 patients, and wedge resection in 9 patients.

In all the patients, the analgesic treatment in the postoperative period included 1% morphine at a rate of 1-2 ml/h i.v., ketoprofen 100 mg i.v., every 12 hours, and metamizol 1 g i.v., every 6 hours.

There were 57 patients (84%) with malignant tumors, including 20 patients (29%) with squamous cell carcinoma, 19 patients (28%) with adenocarcinoma, 6 patients (9%) with lung metastases of the malignant tumors of the other organs, 6 patients (9%) with large cell carcinoma, 4 patients (6%) with adenosquamous carcinoma, one patient (1.5%) with carcinoid, and one patient (1.5%) with lymphoma. Eleven patients (16%) were operated because of nonmalignant tumors, including two patients with tuberculoma, one patient with sarcoidosis, six patients with postinflammatory tumors, and two patients were operated because of hiatal hernia.

Inclusion criterion was as follows: consecutive patients submitted to planned thoracic surgery who gave informed consent for nocturnal, whole-night capnography. Exclusion criteria encompassed patients under 18 years of age, patients who needed opioid treatment in the preoperative period, patients intolerant to ear-lobe sensor, and the patients with severe neuromuscular diseases or thoracic deformities.

Transcutaneous capnography (radiometer TCM4 TOSCA) was performed continuously from 10 p.m. to 6.00 a.m. during the night preceding thoracic surgery in all the patients and repeated during the first night after the surgical procedure in 66 patients. The registration was performed with an ear clip. Transcutaneous oxygen saturation (SpO_2_) and tcpCO_2_ were registered, and the following parameters were analyzed: minimal SpO_2_, maximal SpO_2_, mean SpO_2_, minimal tcpCO_2_, maximal tcpCO_2_, and mean tcpCO_2_. Nocturnal hypoventilation was defined according to American Academy of Sleep Medicine rules, as stated above [7].

The probability of obstructive sleep apnea (OSA) was estimated retrospectively based on the STOP-Bang questionnaire [21] at the end of the follow-up period in all but one alive patient. The score of at least 5 indicated a high risk of moderate-to-severe OSA.

For statistical analyses, program STATISTICA 12 (StatSoft Inc.) was used. The normality of parameters was checked with the Kolmogorov–Smirnov test. Student's *t*-test was used for data comparison. The level of significance of the differences was established at *p* < 0.05.

## 3. Results

Nocturnal hypoventilation in the preoperative period was found in two patients (3%) and persisted in both of them in the postoperative period. Nocturnal hypoventilation in the postoperative period was detected in ten patients (five women). The patients with nocturnal hypoventilation in the postoperative period were all obese (BMI 35.0 ± 4.3 kg/m^2^), three of them had COPD (with mild bronchial obstruction, stage 2) and one of them had daytime chronic hypercapnia in the preoperative period. There were three patients with lung cancer, three patients with lung metastases of renal (two patients) or rectal (one patient) cancers, and four patients with nonmalignant lung tumors.

The patients with postoperative nocturnal hypoventilation as compared with the patients without nocturnal hypoventilation had higher BMI (*p* < 0.05), higher daytime preoperative PaCO_2_(*p* < 0.05), lower SpO_2_ in the preoperative period (*p* < 0.05), higher values of the mean (*p* < 0.01), minimal (*p* < 0.001) and maximal (*p* < 0.0001) tcpCO_2_ in the preoperative period, and higher values of the mean, minimal, and maximal tcpCO_2_(*p* < 0.0001) in the postoperative period; the duration of hospitalization was similar in both groups (Table 1).

The differences in the results of capnographic measurements a night before and a night after thoracic surgery are shown in Table 2. There was a significant increase in the minimal nocturnal tcpCO_2_ and a significant decrease in the mean, minimal, and maximal nocturnal SaO_2_ in the postoperative period as compared with the preoperative period (Table 2).

The mean follow-up of the patients was 26.5 ± 12 months. The STOP-Bang questionnaire was applied to all but one alive patient at the end of follow-up. The mean score in the STOP-Bang questionnaire was 3.5 ± 1.7 and the score ≥5 was obtained in 16 patients (31.4%), all but one obese. Among the patients with postoperative nocturnal hypoventilation, a high probability of moderate-to-severe OSA syndrome as assessed by the STOP-Bang questionnaire was found in four of them.

Twelve patients with primary lung cancer died during the time of observation, including 11 patients without nocturnal hypoventilation in the early postoperative period.

## 4. Discussion

The results of this study with the use of transcutaneous capnography in the patients submitted to thoracic surgery revealed a risk of nocturnal hypoventilation during the first night in the postoperative period in 15% of patients. Nocturnal hypoventilation in the postoperative period occurred exclusively in obese patients and was found in around 22% of the patients with a BMI > 30 kg/m^2^. Coexisting COPD or OSA syndrome also influenced the occurrence of nocturnal hypoventilation in the postoperative period. The results of the diurnal ABG studies did not allow us to predict nocturnal hypoventilation occurring in our patients, although the mean values of preoperative daytime PaCO_2_ were higher in the patient with than in that without nocturnal hypoventilation in the postoperative period, and nocturnal hypoventilation was present in the only patient with chronic daytime hypercapnia in the preoperative period. The differences between tcpCO_2_ and the PaCO_2_ obtained from the ABG studies are usually small and insignificant [22] and may be accepted in almost all the patients with chronic diseases [23]. Thus, it can be admitted that the results of capnography obtained in our patients adequately mirrored respiratory gas exchange abnormalities. Sleep hypoventilation may occur in the patients with normal results of routinely performed daytime ABG studies [8], as in all but one of our patients. Diagnosing sleep hypoventilation is important, as this respiratory abnormality may precede daytime chronic and complete respiratory insufficiency [11].

Sleep hypoventilation in obese persons may develop as a consequence of sleep-breathing disorders, including OSA syndrome [24, 25]. Thus, our patients were asked the questions of the STOP-Bang questionnaire in order to estimate the probability of OSA syndrome, and in two patients with nocturnal hypoventilation, the results were positive.

Transcutaneous capnography in the surgical units was used more than 20 years ago in the pregnant patients during laparoscopic cholecystectomy [26]. In the patients after major colorectal surgery, transcutaneous capnography revealed a significant increase in mean values of tcpCO_2_ measured in the postoperative period as compared with the preoperative period and an even more significant increase in patients receiving opioid analgesia than in the patients receiving epidural anesthesia [27]. In the obese patients undergoing bariatric surgery, the results of transcutaneous capnography showed better agreement with PaCO_2_ than end-tidal capnography [28]. Capnography was successfully applied in the postanesthesia unit in order to evaluate its usefulness in monitoring surgically treated OSA patients [29]. Recently, it has been postulated to incorporate capnography and the STOP-Bang questionnaire [30] or capnography and nocturnal pulse oximetry [31] to the standard practice in the postanesthesia care units in order to reduce respiratory complications after surgical procedures.

In the whole group of our patients, there was a slight but significant decrease of oxygenation of the arterial blood and the increase of the minimal tcpCO_2_ during postoperative night as compared with preoperative night. This could be a result of decreased lung volumes and continuous opioid administration in the early postoperative period. In the smaller group of patients with already established nocturnal hypercapnia, the changes of tcpCO_2_ and SpO_2_ were not significant. Thus, it can be assumed that the analgesic treatment with opioids in standard doses was associated with minimal risk of nocturnal hypoventilation, even in the obese patients. This statement has direct clinical implications, taking into account the decreased pain level in obese patients and thus the need of adequate analgesic treatment [32].

Opioids may induce hypoventilation and other respiratory abnormalities in several ways [33]. Opioids are associated not only with sleep-related hypoventilation but also with OSA [34]. In the patients in the postanesthesia care unit, there is a risk of sustained hypoventilation, which results in hypercapnia, induced by the opioids, as recently shown in the study measuring end-tidal CO_2_ levels and minute ventilation [35]. The other study, however, with the use of capnography in the postanesthesia care unit revealed a tendency to hypocapnia, which in contrary is a result of hyperventilation, and other respiratory abnormalities [36].

The main limitations of the study encompassed the lack of preoperative polysomnography with the evaluation of neurophysiological sleep structure, especially with the possibility of differentiation of REM and non-REM sleep phases, and the detection of sleep-breathing disorders, such as sleep apneas and hypopneas, as well as the lack of postoperative polysomnography. The lack of polysomnography and the ensuing lack of objective data in the presence of sleep-breathing disorders enabled the differentiation between overlap syndromes (COPD + OSA) with or without nocturnal hypoventilation. In addition, if capnography was performed during polysomnography, more information regarding the association of hypercapnia with sleep stages or sleep-breathing disorders could be obtained.

The main strengths of the study are as follows: transcutaneous capnography was performed twice, in the preoperative and postoperative periods in the patients submitted to thoracic surgery; there was a long follow-up period; and the assessment of the coexisting possible sleep-breathing disorder, such as OSA, in the studied population of the patients was performed.

## 5. Conclusion

Nocturnal hypoventilation may occur in the patients after thoracic surgery, especially in obese patients with even mild bronchial obstruction, obstructive sleep apnea syndrome, or chronic daytime hypercapnia.

## Figures and Tables

**Table 1:** The comparison of the patients without (group 0) and with (group 1) nocturnal hypoventilation during the postoperative period.

	*Group 0 n* *=* *58*		*Group 1 n* *=* *10*		*p*
	Mean	SD	Mean	SD	
Age (years)	63.8	7.9	65.1	6.4	NS
Weight (kg)	85.0	19.4	93.3	13.9	NS
BMI (kg/m^2^)	31.0	5.8	35.0	4.2	=0.039
FEV_1_ (l)	2.2	0.8	2.0	0.6	NS
FEV_1_ (% of predicted)	88.1	18.7	85.8	22.8	NS
FVC (l)	3.4	1.1	2.8	0.7	NS
FVC (% of predicted)	105.7	16.5	94.5	24.9	NS
FEV_1_/FVC (%)	67.5	10.3	73.4	8.1	NS
Preoperative PaO_2_ (mmHg)	72.3	6.2	70.5	7.7	NS
Preoperative PaCO_2_ (mmHg)	39.0	3.7	42.0	3.3	=0.023
Preoperative HCO_3_ (mmol/l)	25.2	1.9	25.9	2.5	NS
Preoperative pH	7.42	0.04	7.41	0.03	NS
Preoperative SaO_2_ (%)	94.4	1.5	93.7	2.0	NS
Postoperative PaO_2_ (mmHg)	87.5	22.0	83.6	18.4	NS
Postoperative PaCO_2_ (mmHg)	44.9	4.8	47.9	5.9	NS
Postoperative HCO_3_ (mmol/l)	23.8	1.9	23.4	2.1	NS
Postoperative pH	7.36	0.04	7.35	0.04	NS
Postoperative SaO_2_ (%)	95.1	3.6	94.8	2.0	NS
Preoperative minimal tcpCO_2_ (%)	38.8	3.5	44.8	8.2	=0.0003
Preoperative maximal tcpCO_2_ (mmHg)	47.8	4.6	51.1	10.5	<0.001
Preoperative mean tcpCO_2_ (mmHg)	43.7	3.7	53	5.6	<0.0001
Preoperative minimal tcSpO_2_ (%)	87.8	5.3	4.5	6.5	NS
Preoperative maximal tcSpO_2_ (%)	98.2	1.3	98.2	0.9	NS
Preoperative mean tcSpO_2_ (%)	95.0	1.8	93.7	2.2	=0.037
Postoperative minimal tcpCO_2_ (mmHg)	40.1	3.9	46.4	6.7	<0.0001
Postoperative maximal tcpCO_2_ (mmHg)	49.7	6.4	59.9	8.4	<0.0001
Postoperative mean tcpCO_2_ (mmHg)	44.3	4.3	53.4	6.1	<0.0001
Postoperative minimal SpO_2_ (%)	84.1	7.6	82.7	10.3	NS
Postoperative maximal SpO_2_ (%)	97.3	2.7	96.8	3.2	NS
Postoperative mean SpO_2_ (%)	93.6	5.3	90.5	8.3	NS
Hospitalization (days)	10.9	10.1	9.1	1.9	NS

NS: not statistically significant.

**Table 2:** Comparison of the values obtained in capnography in the whole group during preoperative and postoperative nights in the patients submitted to thoracic surgery.

	*Preoperative*		*Postoperative*		*p*
	Mean	SD	Mean	SD	
Mean tcpCO_2_ (mmHg)	44.4	5.2	45.5	5.9	NS
Minimal tcpCO_2_ (mmHg)	39.6	4.8	40.8	4.8	=0.040
Maximal tcpCO_2_ (mmHg)	49.3	6.9	51.1	7.6	NS
Mean SpO_2_ (%)	94.8	1.9	93.2	5.9	=0.020
Minimal SpO_2_ (%)	87.2	5.6	83.8	8.1	=0.001
Maximal SpO_2_ (%)	98.2	1.2	97.3	2.7	=0.018

NS: not statistically significant.

## Data Availability

The data that support the findings of this study are available from the corresponding author upon reasonable request.

## References

[1] Blüher M. (2019). Obesity: global epidemiology and pathogenesis. *Nature Reviews Endocrinology*.

[2] Mazidi M., Banach M., Kengne A. P. (2018). Lipid and Blood Pressure Meta-analysis Collaboration Group. Prevalence of childhood and adolescent overweight and obesity in Asian countries: a systematic review and meta-analysis. *Archives of Medical Science*.

[3] Abbas A. E. (2018). Surgical management of lung cancer: history, evolution, and modern advances. *Current Oncology Reports*.

[4] Hodgson L. E., Murphy P. B., Hart N. (2015). Respiratory management of the obese patient undergoing surgery. *Journal of Thoracic Disease*.

[5] Mandal S., Hart N. (2012). Respiratory complications of obesity. *Clinical Medicine*.

[6] Böing S., Randerath W. J. (2015). Chronic hypoventilation syndromes and sleep-related hypoventilation. *Journal of Thoracic Disease*.

[7] Berry R. B., Budhiraja R., Gottlieb D. J. (2012). Rules for scoring respiratory events in sleep: update of the 2007 AASM manual for the scoring of sleep and associated events. Deliberations of the sleep apnea definitions task force of the American Academy of sleep medicine. *Journal of Clinical Sleep Medicine*.

[8] Sivam S., Yee B., Wong K., Wang D., Grunstein R., Piper A. (2018). Obesity hypoventilation syndrome: early detection of nocturnal-only hypercapnia in an obese population. *Journal of Clinical Sleep Medicine*.

[9] Piper A. J., Yee B. J. (2014). Hypoventilation syndromes. *Comprehensive Physiology*.

[10] Tondo P., Scioscia G., Hoxhallari A. (2022). Clinical evaluation and management of overlap syndrome (OS) and obesity hypoventilation syndrome (OHS). *Clocks and Sleep*.

[11] McNicholas W. T., Hansson D., Schiza S., Grote L. (2019). Sleep in chronic respiratory disease: COPD and hypoventilation disorders. *European Respiratory Review*.

[12] Sharma S., Stansbury R. (2022). Sleep-Disordered breathing in hospitalized patients: a game changer?. *Chest*.

[13] Masa J. F., Pépin J. L., Borel J. C., Mokhlesi B., Murphy P. B., Sánchez-Quiroga M. A. (2019). Obesity hypoventilation syndrome. *European Respiratory Review*.

[14] Gupta K., Prasad A., Nagappa M., Wong J., Abrahamyan L., Chung F. F. (2018). Risk factors for opioid-induced respiratory depression and failure to rescue: a review. *Current Opinion in Anaesthesiology*.

[15] Nimmo S. M., Foo I. T. H., Paterson H. M. (2017). Enhanced recovery after surgery: pain management. *Journal of Surgical Oncology*.

[16] Gan T. J. (2017). Poorly controlled postoperative pain: prevalence, consequences, and prevention. *Journal of Pain Research*.

[17] Kaw R., Doufas A. G. (2020). Is regular oxygen supplementation safe for obese postoperative patients?. *Cleveland Clinic Journal of Medicine*.

[18] Chindamporn P., Wang L., Bena J. (2022). Obesity-associated sleep hypoventilation and increased adverse postoperative bariatric surgery outcomes in a large clinical retrospective cohort. *Journal of Clinical Sleep Medicine*.

[19] Kaw R., Wong J., Mokhlesi B. (2021). Obesity and obesity hypoventilation, sleep hypoventilation, and postoperative respiratory failure. *Anesthesia and Analgesia*.

[20] Global Initiative for Chronic Obstructive Lung Disease G. O. L. D. (2019). Global strategy for the diagnosis, management and prevention of chronic obstructive lung disease: 2019 report. https://www//goldcopd.org.

[21] Chung F., Abdullah H. R., Liao P. (2016). STOP-bang questionnaire: a practical approach to screen for obstructive sleep apnea. *Chest*.

[22] Fujimoto S., Suzuki M., Sakamoto K. (2019). Comparison of end-tidal, arterial, venous, and transcutaneous P_CO_. *Respiratory Care*.

[23] Maniscalco M., Fuschillo S. (2019). A transcutaneous carbon dioxide monitor is a useful tool with known caveats. *European Respiratory Journal*.

[24] Mokhlesi B., Masa J. F., Brozek J. L. (2019). Evaluation and management of obesity hypoventilation syndrome. An official American thoracic society clinical practice guideline. *American Journal of Respiratory and Critical Care Medicine*.

[25] Perger E., Aron-Wisnewsky J., Arnulf I., Oppert J. M., Redolfi S. (2021). Diagnostic approach to sleep disordered-breathing among patients with grade III obesity. *Sleep Medicine*.

[26] Bhavani-Shankar K., Steinbrook R. A., Mushlin P. S., Freiberger D. (1998). Transcutaneous PCO2 monitoring during laparoscopic cholecystectomy in pregnancy. *Canadian Journal of Anaesthesia*.

[27] McCormack J. G., Kelly K. P., Wedgwood J., Lyon R. (2008). The effects of different analgesic regimens on transcutaneous CO2 after major surgery. *Anaesthesia*.

[28] Liu S., Sun J., Chen X., Yu Y., Liu X., Liu C. (2014). The application of transcutaneous CO2 pressure monitoring in the anesthesia of obese patients undergoing laparoscopic bariatric surgery. *PLoS One*.

[29] Borczynski E., Worobel-Luk P. (2019). Capnography monitoring of patients with obstructive sleep apnea in the post-anesthesia care unit: a best practice implementation project. *JBI Database of Systematic Reviews and Implementation Reports*.

[30] Scully K. R., Rickerby J., Dunn J. (2020). Implementation science: incorporating obstructive sleep apnea screening and capnography into everyday practice. *Journal of PeriAnesthesia Nursing*.

[31] Holt N. R., Downey G., Naughton M. T. (2019). Perioperative considerations in the management of obstructive sleep apnoea. *Medical Journal of Australia*.

[32] Majchrzak M., Brzecka A., Daroszewski C. (2019). Increased pain sensitivity in obese patients after lung cancer surgery. *Frontiers in Pharmacology*.

[33] Davis M. P., Behm B., Balachandran D. (2017). Looking both ways before crossing the street: assessing the benefits and risk of opioids in treating patients at risk of sleep-disordered breathing for pain and dyspnea. *Journal of Opioid Management*.

[34] Rosen I. M., Aurora R. N., Kirsch D. B. (2019). Chronic opioid therapy and sleep: an American Academy of sleep medicine position statement. *Journal of Clinical Sleep Medicine*.

[35] Jungquist C. R., Chandola V., Spulecki C. (2019). Identifying patients experiencing opioid-induced respiratory depression during recovery from anesthesia: the application of electronic monitoring devices. *Worldviews on Evidence-Based Nursing*.

[36] Chung F., Wong J., Mestek M. L., Niebel K. H., Lichtenthal P. (2020). Characterization of respiratory compromise and the potential clinical utility of capnography in the post-anesthesia care unit: a blinded observational trial. *Journal of Clinical Monitoring and Computing*.

